# Examining the relationship between flexible working arrangements and employee performance: a mini review

**DOI:** 10.3389/fpsyg.2024.1398309

**Published:** 2024-07-04

**Authors:** Aydın Çivilidağ, Şerife Durmaz

**Affiliations:** ^1^Department of Psychology, Akdeniz University, Antalya, Türkiye; ^2^Department of Labor Economics and Industrial Relations, Akdeniz University, Antalya, Türkiye

**Keywords:** flexible work arrangements, part-time, telecommuting, compressed work weeks, employee performance

## Abstract

This research aims to determine the relationship between flexible working arrangements (FWAs) and employee performance (EP). The research was conducted by reviewing studies in Web of Science (WoS), EBSCO and Google Scholar databases between 2010 and 2024. The research was screened in the databases in line with the inclusion criteria, which were determined as studies written in English, where data were collected by survey technique, data were analyzed by correlation, and those that met the criteria were included in the research. As a result of the preliminary screening, second screening, and screening in line with the inclusion criteria, the remaining 21 studies constituted the data set of this study. The correlation between FWAs and EP was [*r*_(20)_ = 0.596, *p* < 0.05]. This value can be interpreted a significant and high-level relationship between them. According to the random model, Fisher’s Z and 95% CI (LL = 0.52 and UL = 0.84), Z = 8.45, measured an effect size of 0.35 *p* = 0.000. This value shows a moderate effect size according to Cohen’s d. FWAs have a positive effect on EP, productivity, job satisfaction, job stress, work-family harmony, and organizational commitment. It is recommended that organizations, managers, organizational psychology, and human resources professionals (HRP) should include FWAs in job analysis, job design, and planning.

## 1 Introduction

Flexible working has become increasingly common in many countries in recent years, with many employers offering some form of flexible working to their employees and a significant number of employees taking advantage of these opportunities (De Menezes and Kelliher, 2017). Due to its dynamic nature, working life has been in a state of constant change for the last 50 years. Since the mid-1970s, part-time work has become increasingly common. Part-time work now represents a common form of work arrangements. According to Eurostat, in 2010, around 67% of workers in Europe worked part-time (Devicienti et al., 2018). Especially in the last 25 years, with the widespread use of the internet, computers, and smartphones in social life, working life has changed its form, while it has taken a different dimension with the Coronavirus (Covid-19), which affected the whole world in 2019–2022. The Covid-19 pandemic has led to the reorganization of workplace environment and redesign of work processes in many organizations. In the early times of the pandemic, the number of employees working in enclosed work environments and the operational capacity of organizations were reduced. For full operational use, some organizations waited for the effects of the pandemic to subside, while others took a new approach by taking advantage of technological innovations and passed on to flexible working arrangements (FWA) (Chukwudi et al., 2022). Internet and computer technology initially globalized a significant part of work without time and place limitations. Especially during Covid-19, people worked at workplaces for shorter periods of time (except health workers), while those in some jobs conducted their work from home via the internet. Working from home has become more common during COVID-19 due to public health measures (Zhang et al., 2022). Teleworking, which is different from telecommuting, allows workers to maintain online communication with colleagues and customers even while traveling without the obligation to be tied to a place. Teleworking is defined as a work flexibility arrangement in which an employee carries out the duties and responsibilities related to his/her job at a workplace other than his/her workplace (Kawakubo and Arata, 2022). According to Prasad and Mishra (2021), flexibility in work life is more possible today with the development of technology. Studies have shown that flexible working is positively associated with increased productivity, lower absenteeism, and higher job satisfaction (Scandura and Lankau, 1997; Baltes et al., 1999; Konrad and Mangel, 2000; Kelliher and Anderson, 2008). Since the 2000s, the working arrangement has had to change due to technological developments and epidemics that threaten public health (such as COVID-19, SARS and Bird flu). The most important of these changes is flexible working arrangements. At this point, it is important to determine the effect of flexible working arrangements on employee performance.

### 1.1 Flexible work arrangements

Flexibility in working life gives employees the option to fulfill their work and non-work demands. The term flexible working includes flexibility regarding hours and location and is broad in scope. Shift work, part-time work, telecommuting, compressed work, sabbatical, flexi-time, job sharing, seasonal work, annual working hours, vacation time and much more are related to flexible working (Prasad and Mishra, 2021). Flexi-time is an arrangement that allows employees to choose when to start or finish work, other than regular time, as long as they complete a certain number of hours (Chukwudi et al., 2022). Compressed work weeks are defined as working a full-time schedule while shifting some of those hours to longer days to get more time off on other days. For example, instead of working 8 h a day 5 days a week, working 10 h a day 4 days a week (Galinsky et al., 2011). The term sabbatical (extended leave or personal retreat) has numerous options in today’s ed. context. It can mean taking a break for any time, from a month to several years (Carr and Tang, 2005). Employees have the freedom to choose where they work, which offers significant location flexibility. This means that job duties can be performed from a variety of work locations appropriate to the nature of the job. For example, at home, at the client’s location, on the train, in a café, etc. This type of flexible working arrangement is defined as teleworking (Wessels, 2017). Job sharing is an agreement that allows two or more individuals to work in a full-time job and share responsibilities between themselves (Ifeoma, 2019). According to Aziz-Ur-Rehman and Siddiqui (2019), flexible working helps people to achieve work-life balance. People are committed to both work and personal life and attach importance to both. This helps them to fulfill their work responsibilities as well as their personal life responsibilities while increasing satisfaction in both their personal and professional life. According to Boyer (1988), flexibility and productivity go hand in hand in working life.

### 1.2 Employee performance

According to Güngör (2011), employee performance is what an employee does or does not do. Employee performance includes output quantity, output quality, timeliness of output, presence at work, and cooperation. What organizations expect from employees is to do the work fully and accurately. Task performance and contextual performance are determinants of employee performance. Task performance is the employee’s fulfillment of the responsibilities in the job description. Contextual performance refers to behaviors that are not directly related to one’s job, but voluntarily support the success of others for the effectiveness of the organization (Sarpkaya and Bayraktar, 2023). According to Prasad and Mishra (2021), employee performance is a key factor for an organization to survive in competition. Individual performance plays an important role in a higher level of organizational performance. An individual’s high performance in performing their tasks results in feelings of satisfaction, self-efficacy, and mastery. Employee performance refers to the achievement of agreed work outcomes on the employee’s work behaviors, which can be achieved through productivity, work quality, or other means (Ludidi, 2020). Although there have been many studies on the impact of flexible working arrangements on employees’ work behaviors and performance, this research was conducted because there were not enough meta-analysis studies that addressed the relationship between flexible working arrangements and, the Covid-19 process and, technological changes. This research aims to determine the relationship between flexible working arrangements and employee performance between 2010 and 2024. In line with this purpose, the research questions are given below:

1. What are the concepts in the studies on flexible working arrangements and employee performance?
2. What is the relationship and the effect size between flexible working arrangements and employee performance in the studies included in this research?

Due to technological developments in 2010–2024 and COVID-19 changing working arrangements, only studies during this period were included in the scope of this research.

## 2 Method

This research was conducted by systematic review method and its procedure. This research aims to construct a mini review on flexible work engagements and employee performance. The research is based on published studies examining the relationship between flexible working arrangements and employee performance between 2010 and 2024 years and follows the PRISMA method of systematic review (Moher et al., 2009).

### 2.1 Data collection process

The research data were searched in WoS, EBSCO and Google Scholar databases by entering “and,” “or” between the keywords “Flexible working arrangements,” “Employee performance,” “Job performance,” “Employee productivity,” “Flexible working,” “Worker performance,” “Flexi time.” Research articles and theses written in English, in which correlation analysis was applied among the studies that collected data with survey method between 2010–2024 were used as inclusion criteria. Master’s and doctoral theses require the application of scientific research methods and techniques. In addition, thesis must be approved by a jury of at least three academics in order to be accepted. For this reason, thesis are accepted as an academic and scientific work. All studies other than research articles were excluded. The titles, abstracts, and method sections of the 884 studies identified in the initial screening were read one by one by the researchers, and 101 studies that could be included in the scope of the study were identified. Of these studies, 21 studies that were not suitable for the research focus were eliminated, the full texts of the remaining 80 studies were read in detail by two researchers separately to determine whether they fully fulfilled the inclusion criteria, and the remaining 21 studies (18 articles, 2 master thesis and 1 doctoral thesis) were determined to meet the research criteria by consensus between the two authors.

### 2.2 Data analysis

CMA 3.0 program was used to calculate the effect size between variables. The authors examined the 21 studies included in the study in detail one by one. Differences of opinion between the researchers during the data analysis were resolved by making joint decisions. Accordingly, the authors, study type, location, sample size, participants’ profession, language, data collection method, study variables, and number of citations were determined. Accordingly, the total sample size of the studies conducted in 9 different countries is 4,274. Other characteristics of the studies are presented in Table 1 below.

**Table 1:** Characteristics of the studies.

**Id**	**Authors**	**Study type**	**Country**	***N***	**Participants**	**Language**	**Correlation coefficient**	**Scales**	**Cited by**
1	Agbanu et al. (2023)	Article	Nigeria	162	Sales Representatives	English	0.798*	FWAS, EProS.	3
2	AhmedAlqasa and Alsulami (2022)	Article	Saudi Arabia	107	Education Sector Employees	English	0.460**	FWOQ, EPS	1
3	Altındağ and Siller (2014)	Article	Türkiye	200	Various Sector Employee	English	0.207*	FWMS, EPS	69
4	Alwi (2021)	Article	Pakistan	Unspecified	Multinational Company Employees	English	0.412**	FWQ, EPS	0
5	Babu et al. (2011)	Article	India	300	IT Employees	English	0.589**	FTS, EPS	0
6	Bett et al. (2022)	Article	Kenya	137	Agricultural Cooperatives Employees	English	0.801**	FWAS, EPS	0
7	Dikirr and Omuya (2023)	Article	Kenya	260	Academic Staff	English	0.467**	FTQ, EPQ	1
8	Govender (2017)	PhD. Thesis	South Africa	92	Unspecified	English	0.585**	FWAS, EP	5
9	Mbae et al. (2019)	Article	Kenya	219	Electricity Company Employees	English	0.753**	JFS, EPS	1
10	Muga and Senelwa (2022)	Article	Kenya	155	Public Health Sector	English	0.088	FWPS, EPS	1
11	Mwebi and Kadaga (2015)	Article	Kenya	291	Bank Employees	English	0.344**	FWAQ, EPQ	37
12	Nayanathara and Karanurathne (2021)	Article	Sri Lanka	169	IT Employees	English	0.521**	FWS, EJP	2
13	Nnko (2022)	Ph.D. Thesis	Kenya	404	Nurse	English	0.469**	WSS, NPS	1
14	Nurudeen et al. (2024)	Article	Nigeria	248	Private Hospital Employee	English	0.711**	FWAQ, EPQ	0
15	Obisi (2017)	Article	Nigeria	160	School Employee	English	0.720**	FWSS, EPS	11
16	Odunayo and Kelvin (2020)	Article	Nigeria	285	Logistics Companies Employee	English	0.921**	FWOQ, EPQ	11
17	Osisioma et al. (2016)	Article	Nigeria	33	Health Workers	English	0.411*	FWHS, EPS	11
18	Ramlan et al. (2023)	Article	Malaysia	60	Bank Employees	English	0.828**	FWQ, JPQ	0
19	Sekhar and Patwardhan (2023)	Article	India	214	Service Firm Employees	English	0.299**	TFWAS, JPS	19
20	Tamunomiebi (2018)	Article	Nigeria	327	Academic Staff	English	0.675**	FTWPQ, EPQ	6
21	Yau (2023)	Master Thesis	Malaysia	201	Private Sector	English	0.529**	FWAS, EPS	0

*p < 0.05, **p < 0.01.FWAS, Flexible Work Arrangement; EProS, Employee Productivity; FWOQ, Flexible Work Options questionnaire; EPS, Employee Performance; FWMS, Flexible Working Method Scale; FWQ, Flexible Working Questionnaire; FTS, Flexi Time Scale; FWAS, Flexible Work Arrangement Scale; FTQ, Flexi Time Questionnaire; EPQ, Employee Performance Questionnaire; JFS, Job Flexibility Scale; FWPS, Flexibility Work Practice Scale; FWAQ, Flexitime Work Arrangement Questionnaire; FWS, Flex Work Scale; EJP, Employee Job Performance; WSS, Work Scheduling Scale; NPS, Nurses Performance Scale; FWAQ, Flexible Work Arrangements Questionnaires; FWSS, Flexible Work Schedules Scale; FWHS, Flexible Working Hours Scale; JPQ, Job Performance Questionnaire; TFWAS, The Flexible Working Arrangement Scale; JPS, Job Performance Scale; FTWPQ, Flexi Time Work Practice Questionnaire.

## 3 Findings

The 21 studies within the scope of the research were conducted in 9 different countries. When these countries are analyzed, 6 studies were conducted in Kenya and 6 studies were conducted in Nigeria, 13 of which are located in the African continent, while 1 study was conducted in South Africa. In Asia, 7 studies were conducted, 1 in Saudi Arabia, 2 in India, 1 in Pakistan, 1 in Sri Lanka, and 2 in Malaysia. From the European continent, there is only 1 study conducted in Turkey. The total sample size of these studies is 4,274. The studies included participants from a variety of different business areas (such as health, education, banking). In the studies, Likert-type scales are used to measure flexible working arrangements and employee performance. Employee performance is generally measured with the employee performance scale. Measurement tools such as flexible working options scale, flexi time questionnaires, flexibility work practice scale, flexible work schedules scale are used to measure flexible working arrangements. When the studies are analyzed according to their citations, it is seen that the 3rd-ranked study conducted in Turkey is the most cited. The high citation rate of this study may be due to the fact that it was published earlier than other studies. This study was followed by the study conducted in Kenya, which ranked 11th and received 37 citations. Apart from these, the study conducted in India in 2011, which ranked 6th, did not receive any citations. Likewise, studies ranked 4th, 5th, 6th, 18th, and 21th did not receive any citations. It should be noted that these studies are more recent than the 2011 study. According to the heterogeneity and effect size calculations, the random effect model is used since the heterogeneity value is *p* = 0.000 and *p* < 0.05. Also Q = 544.469, df = 20 is found from FWAs-EP values. Chi-square for FWAs-EP is 31.410 at 0.05 level of significance at 20 degrees of freedom. The *I*^2^ value is calculated as 96, 327%, which is greater than 75% in the *I*^2^ indicator, thus indicating high heterogeneity among the research variables (Borenstein et al., 2021). Random model is used since the data showed heterogeneity. According to the random model, the correlation between FWAs and EP is [*r*_(20)_ = 0.596, *p* < 0.05]. The correlation value corresponding to Fisher’s *Z* indicates a statistically high, significant and positive relationship. The effect size [Fisher’s *Z* and 95% CI (LL = 0.52 and UL = 0.84), *Z* = 8.45] is measured 0.35 and *p* = 0.000. According to Cohen’s *d*, this value can interpret a moderate effect size (Cohen, 1988).

**Figure 1:**
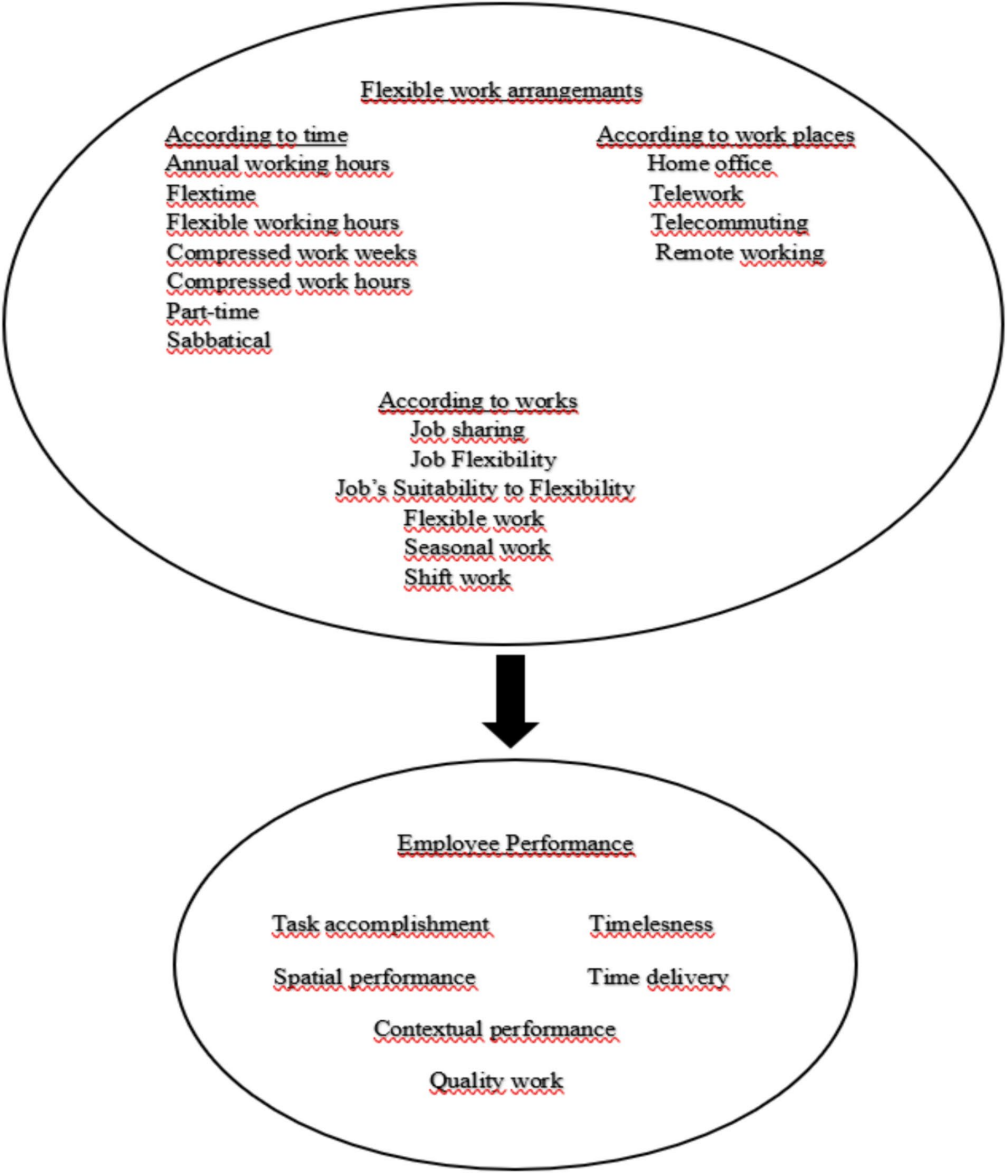
Conceptual framework.

Flexible working arrangements can be made in three contexts: time, works and workplaces. Employee performance is about getting things done successfully, on time and with good work quality. Figure 1 below shows the impact of flexible working arrangements on employee performance.

## 4 Conclusion and recommendations

According to the results of meta-analysis applied to research data obtained after a systematic review of studies; the mean correlation value of flexible working arrangements and employee performance is calculated as [*r*_(20)_ = 0.596]. Accordingly, it can be said that there is a highly significant and positive relationship between flexible working arrangements and employee performance. The calculated effect size is 0.355. This value indicates a moderate effect size according to Cohen’s *d*. Therefore, in line with these results, it can be said that flexible working arrangements have a significant and moderate effect on employee performance. This result is consistent with many studies (Giovanis, 2018; Ifeoma, 2019; Ramakrishnan and Arokiasamy, 2019; Idowu, 2020; Ludidi, 2020; Msuya and Kumar, 2022; Mustakim, 2022; Yuliati et al., 2023). Organizations can offer flexible working arrangements to retain productive employees. This has been shown to increase employee’ performance, satisfaction, loyalty, and productivity, and reduce absenteeism and recruitment costs (Giovanis, 2015; Abid and Barech, 2017; De Menezes and Kelliher, 2017; Bukhari et al., 2018). Flexible working arrangements are beneficial for employees in terms of reducing work stress, increasing job satisfaction and life satisfaction, providing mental and physical balance, and for organizations in terms of increasing work efficiency and effectiveness (Costa et al., 2006; Masuda et al., 2012; Solanki, 2013; Kröll and Nüesch, 2017). Flexible working arrangements help employees to manage their workload, personal life, and responsibilities. They also reduce conflict by helping employees better manage the boundaries between work and home life (Jivan, 2017; Aziz-Ur-Rehman and Siddiqui, 2019; Eshak et al., 2021; Chukwudi et al., 2022). Research generally reveal the positive effects of flexible working arrangements on employee behavior and performance. More specifically, employees with flexible working arrangements can control work stress caused by tension, fatigue and excessive workload. Burnout and work accidents caused by intense and excessive work tempo can be prevented. Conflicts between employees and their colleagues or supervisors can be reduced with flexible working arrangements. Since employees can arrange their work hours according to their needs and the demands of their families, they may have positive feelings towards their work and the organization, which may reflect positively on their job performance. Employees who are allowed to work flexibly generally show increased organizational commitment, attendance, and performance. Commitment to the team was seen as a prerequisite for flexible working to be effective as well as for strengthening organizational commitment. Organizations with flexible working arrangements can provide more versatility to their employees and thus create a more compatible work pattern than organizations with fixed work patterns and working hours (Altındağ and Siller, 2014; Choudhary, 2016; Mungania et al., 2016; Ifeoma, 2019; Anya et al., 2021).

The results of this research can be used especially by managers, organizational psychology and HRP for job analysis, job design, job planning, job motivation, and performance appraisal. Flexible working arrangements can protect employees against stress, especially from the work itself, customer intensity, extreme hot, cold or humid working conditions, and family-work harmony. The research is limited by the number of studies included in the systematic review. Further studies in different cultures in Europe, which are not included in this research in sufficient numbers, and the Americas and Australia, which are not included in this research, may reveal more comprehensive results. To better understand the psychological, social, cultural, and economical effects of flexible working, it is recommended to research employees and organizations in different sectors in different countries.

## Author contributions

AÇ: Conceptualization, Data curation, Formal analysis, Funding acquisition, Investigation, Methodology, Project administration, Resources, Software, Supervision, Validation, Visualization, Writing – original draft, Writing – review & editing. ŞD: Conceptualization, Data curation, Formal analysis, Investigation, Methodology, Resources, Validation, Visualization, Writing – original draft, Writing – review & editing.
